# An integrated community and primary healthcare worker intervention to reduce stigma and improve management of common mental disorders in rural India: protocol for the SMART Mental Health programme

**DOI:** 10.1186/s13063-021-05136-5

**Published:** 2021-03-02

**Authors:** Mercian Daniel, Pallab K. Maulik, Sudha Kallakuri, Amanpreet Kaur, Siddhardha Devarapalli, Ankita Mukherjee, Amritendu Bhattacharya, Laurent Billot, Graham Thornicroft, Devarsetty Praveen, Usha Raman, Rajesh Sagar, Shashi Kant, Beverley Essue, Susmita Chatterjee, Shekhar Saxena, Anushka Patel, David Peiris

**Affiliations:** 1 The George Institute for Global Health, New Delhi, India; 2 University of New South Wales, Sydney, Australia; 3 Prasanna School of Public Health, Manipal, India; 4 The George Institute for Global Health, Oxford, UK; 5 The George Institute for Global Health, Hyderabad, India; 6 The George Institute for Global Health, University of New South Wales, Sydney, Australia; 7 Centre for Global Mental Health and Centre for Implementation Science, Health Service and Population Research Department, Institute of Psychiatry, Psychology and Neuroscience, King’s College London, London, UK; 8 University of Hyderabad, Hyderabad, India; 9 All India Institute of Medical Sciences, New Delhi, India; 10 Institute for Health Policy, Management and Evaluation, University of Toronto, Toronto, Canada; 11 Harvard T H Chan School of Public Health, Boston, USA

**Keywords:** Common mental disorders, Primary healthcare worker, Anti-stigma campaign, Electronic decision support systems, SMART Mental Health, India, Implementation, Cluster randomised controlled trial

## Abstract

**Background:**

Around 1 in 7 people in India are impacted by mental illness. The treatment gap for people with mental disorders is as high as 75–95%. Health care systems, especially in rural regions in India, face substantial challenges to address these gaps in care, and innovative strategies are needed.

**Methods:**

We hypothesise that an intervention involving an anti-stigma campaign and a mobile-technology-based electronic decision support system will result in reduced stigma and improved mental health for adults at high risk of common mental disorders. It will be implemented as a parallel-group cluster randomised, controlled trial in 44 primary health centre clusters servicing 133 villages in rural Andhra Pradesh and Haryana. Adults aged ≥ 18 years will be screened for depression, anxiety and suicide based on Patient Health Questionnaire (PHQ-9) and Generalised Anxiety Disorders (GAD-7) scores. Two evaluation cohorts will be derived—a high-risk cohort with elevated PHQ-9, GAD-7 or suicide risk and a non-high-risk cohort comprising an equal number of people not at elevated risk based on these scores. Outcome analyses will be conducted blinded to intervention allocation.

**Expected outcomes:**

The primary study outcome is the difference in mean behaviour scores at 12 months in the combined ‘high-risk’ and ‘non-high-risk’ cohort and the mean difference in PHQ-9 scores at 12 months in the ‘high-risk’ cohort. Secondary outcomes include depression and anxiety remission rates in the high-risk cohort at 6 and 12 months, the proportion of high-risk individuals who have visited a doctor at least once in the previous 12 months, and change from baseline in mean stigma, mental health knowledge and attitude scores in the combined non-high-risk and high-risk cohort. Trial outcomes will be accompanied by detailed economic and process evaluations.

**Significance:**

The findings are likely to inform policy on a low-cost scalable solution to destigmatise common mental disorders and reduce the treatment gap for under-served populations in low-and middle-income country settings.

**Trial registration:**

Clinical Trial Registry India CTRI/2018/08/015355. Registered on 16 August 2018.

**Supplementary Information:**

The online version contains supplementary material available at 10.1186/s13063-021-05136-5.

## Contributions to literature

- Mental health services in low- and middle-income countries (LMICs) are poor, and only about 4–5% of those who suffer from disorders such as depression receive appropriate care
- This project uses a combination of task sharing, anti-stigma campaign and technology-enabled mental health service delivery model for rural India for treating community members suffering from stress, depression and increased suicide risk.
- The anti-stigma campaign will be the largest such programme in a low- and middle-income country.
- The SMART Mental Health project will inform the government about strategies that might be beneficial for addressing the gaps in providing care for those suffering from mental disorders not only in India but also in other LMICs too.

## Background

### Common mental disorders: burden and treatment gap

The 2016 National Mental Health Survey in India estimated that the prevalence of any mental illness among adults is about 15%, with nearly 150 million people in need of treatment [1]. It is also reported that one in seven individuals are affected by mental disorders [2]. The prevalence of depression, anxiety and substance use disorders accounts for the majority of mental illness and is estimated to be around 10% [1]. Further, earlier research has shown that India has relatively high suicide rates [3]. There are high levels of variation across India with Andhra Pradesh having one of the highest suicide rates in the country at around 37.5 per 100,000 population [4]. In our previous work, we found that the prevalence of common mental disorders (CMDs)—which include depression, anxiety and suicidality (elevated suicide risk)—in rural Andhra Pradesh was estimated to be around 5% [5]. In addition to the personal and familial costs, one study estimated the reduction in economic growth attributable to mental illness in India and China would be greater than USD 9 trillion between 2016 and 2030 [6].

The treatment gap (i.e. the difference between number of people with a mental disorder and the number receiving appropriate care) for people with CMDs is large—estimated to be 75–95% in India, and further gaps in the quality of care are even more marked with only around 4% of people with major depressive disorders receiving guideline-recommended care [6–9]. The reasons for these gaps are multifactorial, and demand-side barriers include limited awareness among and identification of individuals with mental illness, stigma, discrimination and negative community perceptions about mental health and help seeking [10, 11].

Supply-side barriers include a lack of trained mental health professionals and availability of specialist services [12, 13]. The rate of psychiatric health care professionals in high-income countries is approximately 75 to 120 times greater than the rates seen in India [13, 14] with the density of health care staff much lower in rural areas.

### Primary mental healthcare challenges

Given the large access and treatment gaps in addressing CMDs, India’s health system, particularly at the primary health care level, faces great challenges in addressing this shortfall. The National Mental Health Programme has defined strategies to enhance mental health services; however, implementation in rural regions is grossly limited by workforce constraints. India’s three-tier healthcare system provides nurse/midwife-level primary healthcare at the sub-centre level, covering a population of approximately 3000 to 5000; doctor-level care at the primary health centre (PHC) catering to approximately 20,000 to 30,000 population; and specialised care at the community healthcare centres covering approximately 80,000 to 120,000.

Given the low availability of specialty-trained professionals, alternative solutions which involve the delivery of mental healthcare by primary care physicians and community health workers hold promise and there is emerging evidence that basic mental healthcare can be provided by trained non-physician health workers [15, 16]. It is therefore imperative to develop innovative strategies to increase access to a basic standard of guideline-informed mental healthcare.

The Indian government contracts community health workers known as accredited social health activists (ASHAs) in each village with one ASHA servicing a population of about 1000 individuals. ASHAs are recruited by the Panchayat (local village-level government) and are female residents of the village who are generally educated to grade 8–10 level, although it may be much lower in remote areas. They are provided basic training in health services delivery and their primary role is to support the government programme around maternal and child health on a part-time (2–3 h per day) contractual basis. One potential way to help bridge the mental health care gap in rural areas in India is to utilise the services of ASHAs. However, such an approach requires scalable strategies for training and provision of support tools for all relevant healthcare workers.

### Mobile health (mHealth) strategies support primary healthcare

Mobile health (mHealth) technologies have potential to address access and treatment gaps for common health conditions in primary healthcare settings. One potential mHealth application to strengthen workforce capacity is the use of electronic decision support system (EDSS) which could be deployed in rural communities. Integration of EDSS into mobile devices (mobile phones, smartphones and tablets) can increase reach due to the growing penetration of mobile devices, including smart phones, in Indian communities [17, 18]. The potential benefits of an EDSS in managing a number of health conditions, including mental health, have been demonstrated mainly in high-income country settings, particularly for tools with evidence-based algorithms that provide individualised advice at the point of care [19, 20]. However, trials of such tools have mainly resulted in improved processes of care, with relatively fewer studies demonstrating improvements in clinical outcomes [21]. In India, a recent systematic review of mHealth interventions found fragmented and low-quality evidence on the efficacy, acceptability, and cost-effectiveness of such initiatives [22].

### SMART Mental Health—anti-stigma campaign and EDSS development

In order to address these research gaps, our aim is to develop and evaluate a complex intervention incorporating an anti-stigma strategy and a mobile-technology-based EDSS to facilitate delivery of mental health care for CMDs in rural India—the Systematic Medical Appraisal, Referral and Treatment (SMART) Mental Health Programme. It builds on pilot work conducted in one south Indian state, Andhra Pradesh [23], and has since been expanded for implementation in north India (Haryana state).

### Anti-stigma campaign materials

Three strategies have been developed to address mental health-related stigma: educational materials that target inaccurate knowledge and stereotypes, interpersonal contact with members of a stigmatised community and public protests against those who stigmatise other groups [24]. Reviews of anti-stigma campaigns have shown that interpersonal contact and to a lesser degree educational materials have been variably effective, with most evidence coming from high-income countries [24, 25].

The anti-stigma materials were developed for the project in Andhra Pradesh and then adapted for use in Haryana. Formative research was conducted to inform the development of information, education and communication (IEC) materials drawing on earlier research and adaption to local context [26]. The content includes the following:

1. Printed IEC materials: brochures, flipbooks, pamphlets, calendars and posters on signs and symptoms of CMD such as depression, anxiety, substance use and suicide risk; the need for seeking treatment and treatment/management options apart from medicines and psychotherapy; and issues of stigma related to mental health prevalent in the community. Vignettes and simple illustrations on CMDs will be included in the brochures as examples. Brochures and flipbooks are used during door-to-door campaigns and community meetings to raise mental health awareness and discuss issues related to stigma. ASHAs, auxiliary nurse midwives (ANMs), field staff and PHC doctors will be involved in distributing these materials. The posters and pamphlets can also be shared with local government or panchayat offices, schools and primary health centres and displayed on their walls or notice boards.
2. Involving a person with CMD to talk about his/her experience: video narratives from a person with CMD and a caregiver who discusses their experiences of mental illness and treatment. This video can be screened and discussed as part of the anti-stigma campaign.
3. Promotional and awareness videos on mental health, stigma and the SMART Mental Health project: these have been developed using local film actors from both regions who speak about CMD and this content can also be screened during the campaign phase of the programme.
4. Short animation videos: Two short animations of about 90 s each were developed focussing on stigma concepts proposed by Thornicroft et al. [27].
5. Staging a skit by a local theatre group: A theatrical skit on domestic violence, depression and the need for being treated has been developed. Two theatre groups have been employed to enact the scripts in the two regions (Andhra Pradesh and Haryana). The script has been designed to complement the information in the IEC materials. Live performances in the village can be staged and video recordings of the entire play and short segments can be screened in villages in addition to or in place of live shows.

The above strategies were designed to address lack of awareness and misinformation about mental health and used education-based, interpersonal and social contact approaches to increase knowledge and reduce stigma, discrimination and social avoidance related to CMDs. Content was developed in Telugu and Hindi, the most commonly spoken languages in the two regions of the study.

### mHealth-based EDSS

Separate EDSS modules were developed for ASHAs and PHC doctors. The software was developed on an Android platform, optimised for 7-in. tablets. The EDSS for ASHAs included a screening tool based on the Patient Health Questionnaire (PHQ-9) [28, 29] and Generalised Anxiety Disorders (GAD-7) instruments [29, 30]. Both these scales have been validated in India [31]. The diagnostic and management guidelines used by the PHC doctor are based on WHO’s Mental Health Gap Action Programme-Intervention Guide (mhGAP-IG) [32] and focus on three conditions: depression, anxiety and self-harm/suicide. The mhGAP-IG is in English. The algorithm has been modified based on multiple iterations and feedback from the research team and a psychiatrist and has been validated in a previous mixed methods study [26]. The treatment algorithm provides guidelines about both pharmacological and psychological treatment including referral. A traffic-light dashboard was also developed to support ASHAs to follow-up screen-positive individuals and act on the treatment provided by the doctors.

The implementation of the enhanced mobile technology-based EDSS for ASHA and PHC doctors to identify and treat people at high risk of CMDs includes the following:

1. Non-physician health workers (ASHAs) are trained to use the PHQ-9 and GAD-7 to screen adults (≥ 18 years) in the village for CMDs. They use a tablet-based questionnaire to generate composite scores to identify ‘high-risk’ individuals.
2. ASHAs can then provide basic supportive advice and a referral to the PHC doctor. Participants are electronically referred via the mHealth platform and provided with a paper referral card to take to the doctor.
3. The PHC doctor then reviews participants as part of a village health camp visit or the patient can directly visit the PHC. Data from individuals are uploaded, with consent, to a secure health record using the OpenMRS open source, electronic health record system.
4. A decision support application based on WHO’s mhGAP-IG algorithm is integrated in a tablet device and used by PHC doctors to diagnose and manage patients experiencing CMDs. Doctors can use the depression, self-harm/suicide and emotional or medically unexplained complaints’ modules of mhGAP-IG. Patients requiring medication can access these directly from the PHC or purchase low-cost generics from a private pharmacy.
5. For people with complex needs, consultation with a specialist psychiatrist is available as needed. The psychiatrist can also conduct case reviews via phone and/or video conferencing with the PHC doctors to enhance their management skills. Alternatively, people can be referred by the PHC doctor to a psychiatrist in the next tier of the public health system.
6. Mobile-based messages are sent to ASHAs to ensure adherence and follow-up of those individuals who are diagnosed with CMDs by PHC doctors. ASHAs are able to conduct follow-up visits and assess treatment adherence using a recall and reminder (priority listing) system installed on their mobile devices. ANMs will also assist in streamlining the referral system by ensuring that patients under treatment are regularly followed-up.
7. An algorithm-based interactive voice response system is able to send out pre-recorded messages to diagnosed patients to continue care as advised by the PHC doctor and ASHA, and similar motivational messages can be sent to ASHAs to screen and follow up patients as per guidelines.

## Research methods

### Study objectives

The study aims to evaluate the feasibility, clinical effectiveness and cost-effectiveness of a multifaceted primary healthcare worker intervention to identify and manage CMDs. The study hypothesises that SMART Mental Health will address mental healthcare access barriers and lead to significant improvements in community behaviours toward mental disorders, and improvements in the proportion of adults at high risk of CMDs achieving remission for depression, anxiety and suicide risk.

### Study design

A parallel-group cluster randomised, controlled trial (cRCT) involving PHCs in rural Andhra Pradesh and Haryana will be conducted. The adult population will be screened to identify two cohorts: (1) people at ‘high-risk’ of CMDs and (2) a random sample of people not at high risk at baseline. These will comprise the evaluation cohorts and they will be followed up for 12 months. It is a hybrid type 2 implementation trial where the primary outcome is based on the equivalence of these two cohorts. Detailed process and economic evaluations will also be conducted. An additional post-trial sustainability assessment phase will be implemented where the intervention will be offered to both trial arms for as part of a non-randomised evaluation. In this phase, there will be less intensive monitoring and support provided of primary health workers, in order to assess implementation in a non-research setting and to explore potential for scale-up.

### Study schema

**Fig. 1:**
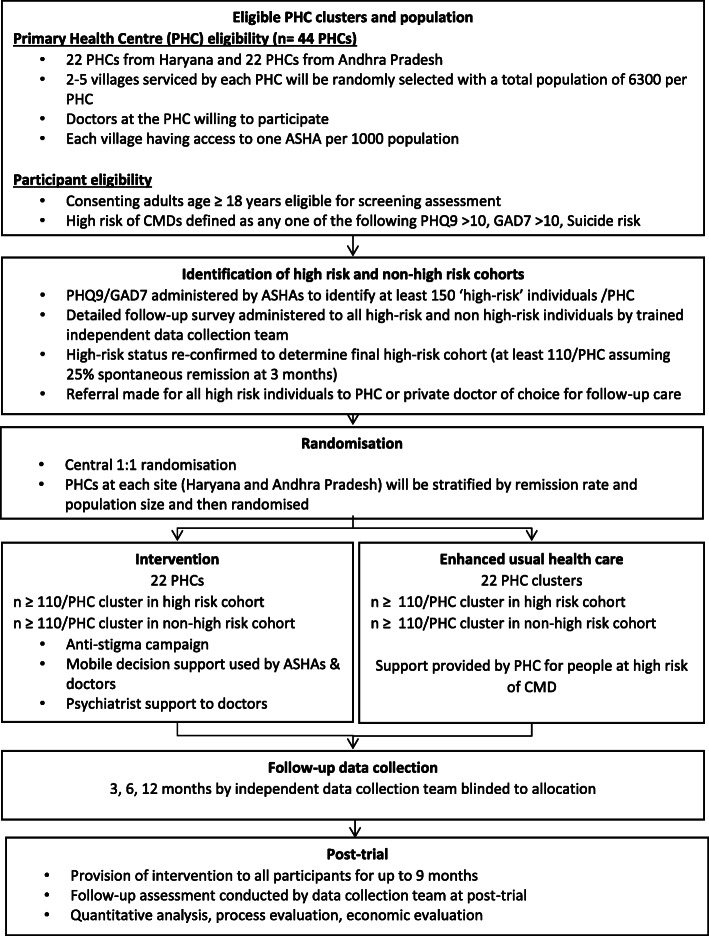
Study schema

Figure 1 outlines the study schema.

### Study population and sites

**Fig. 2:**
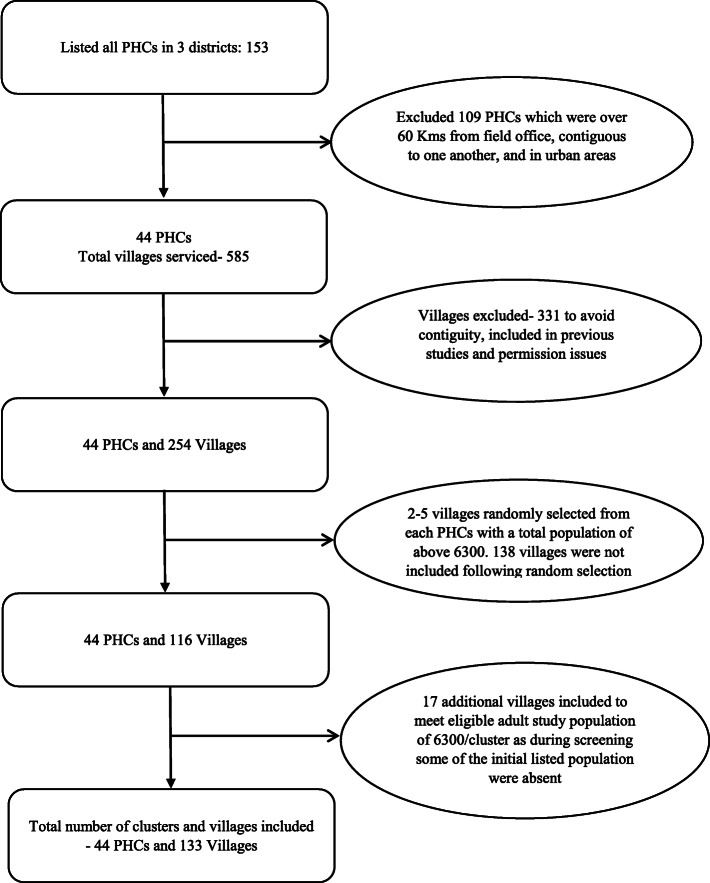
Site selection

A total of 44 PHCs servicing 133 villages in West Godavari district of Andhra Pradesh, and Faridabad and Palwal districts of Haryana, will take part in the study. The eligibility criteria for PHCs and villages are indicated in Fig. 1, and the steps in the selection of the sites are detailed in Fig. 2.

### Eligible population

All consenting adults ≥18 years of age will be eligible for screening for CMDs to identify both a high-risk and non-high-risk cohort. ASHAs will be trained to screen adults in their villages.

#### High-risk cohort

Up to 165,000 adults will be screened until at least 150 individuals per PHC at high risk of CMDs have been identified. High risk is defined as the presence of at least one of the following:

1. High risk of depression based on PHQ-9 score ≥ 10.
2. High risk of anxiety based on GAD7 score ≥ 10.
3. Positive response (score ≥ 2) to the suicide risk question on the PHQ-9.

Given that there may be some time delay between screening and randomisation (at least 12 weeks), these participants will be rescreened to assess if they still meet the high-risk inclusion criteria prior to randomisation. The literature suggests that around 25% of people initially identified at high risk will no longer meet the high-risk criteria at 3 months [33], and consequently, we expect the final high-risk cohort to consist of at least 110 individuals per PHC.

#### General population cohort

A second cohort of 110 adults per PHC not at high risk for CMD will be identified by selecting a random sample from the remaining screened population. This will form the ‘non-high-risk’ cohort.

### Exclusion criteria

Participants with either severe physical or mental ill health that would prevent regular follow-up will not be included in the study.

### Randomisation

Cluster randomisation will occur at the level of the PHC. A total of 44 PHCs from West Godavari district of Andhra Pradesh and Faridabad and Palwal districts of Haryana will be selected. Allocation of PHCs (including 2–5 villages per PHC) to intervention or control arm will use a computer-generated 1:1 central allocation sequence. Random allocation for PHC will be performed using SAS PROC plan or another relevant procedure. PHCs at each site will be stratified by natural remission rate after the initial screening is completed, and population size of the PHCs. To maintain blinding, the unblinded statistician will generate the actual randomisation list and share with field staff. The blinded statistician on the study will work with dummy treatment arms until the end of study. The treatment-related columns will not be shared with blinded statistician/statistical team until the time of planned study unblinding.

### Intervention components

**Fig. 3:**
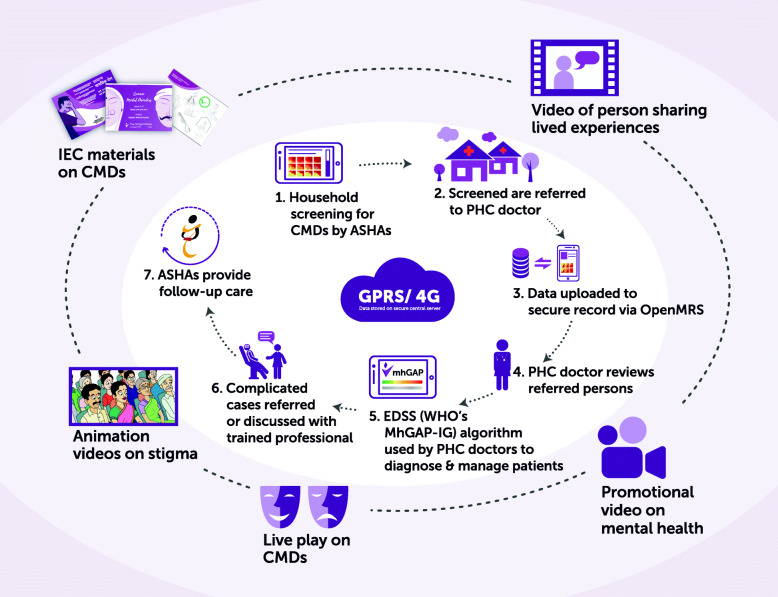
Intervention prototype of SMART Mental Health programme

The two core intervention components of the SMART Mental Health programme have been described above and include the community anti-stigma campaign and electronic decision support system. Figure 3 outlines how the different components of the trial work together. The anti-stigma campaign in particular will have an intensive 3-month phase at the beginning where the aim will be to implement all the components of the campaign. This will be followed by repeat roll out of individual components of the anti-stigma campaign over the next few months of the intervention in a staggered manner.

### Control arm

All participating PHCs (including those randomised to the control arm) and villages will receive information on mental health and common mental disorders and the importance of help seeking in the form of brochures and pamphlets. Those identified at high-risk will be informed about their risk and advised to seek care from a PHC doctor or psychiatrist by the ASHAs. The PHC doctors will be informed that some patients with CMDs may seek care and could be treated at the PHCs or referred to specialists as per current practice. Any patient displaying psychotic symptoms, elevated self-harm risk or other symptoms that potentially reflect serious mental illness other than depression and anxiety will be referred to the nearest health facility where a psychiatrist is available.

### Both arms

For all participating PHCs, we will liaise with government and private pharmacies to ensure availability of appropriate medications. We will also explore collaborative strategies with the Ministry of Health and Family Welfare to enhance the availability of common psychotropic medications as per the Essential Drugs List in the PHCs.

### Data collection

Independent field investigators, blinded to intervention allocation, will be involved in data collection at each phase of the study. Data collection will take place on five occasions for each of the intervention and control PHCs: prior to randomisation (time 0), during intervention (3, 6, 12 months) and at the end of the post-trial phase. All data including clinical data of study participants will be captured on tablets, de-identified and saved in a secure server.

- Time—0 month (pre-randomisation): a detailed questionnaire will be administered among the high-risk and non-high-risk cohort that will enquire about sociodemographic characteristics, treatment history, past history of any mental illness, family history of any mental illness, social support from friends and families, stressful events experienced in the last 1 year, history of any comorbid major physical illnesses, quality of life and costs incurred on treatment.
- Time—3, 6 and 12 months (intervention phase): Data will be collected from both high-risk and non-high-risk cohorts across all the PHCs by trained interviewers blinded to intervention allocation. Questionnaires will administer the PHQ-9, GAD7, Knowledge, Attitude, and Behaviour (KAB) [34] and Barriers to Access to Care Evaluation-Treatment Stigma (BACE-TS) [35] at these time points.
- Following the cRCT, both the control and intervention arm will continue to receive the anti-stigma and mHealth components for up to 9 months. Subsequently, both high-risk and non-high-risk adults will be asked questions using PHQ-9, GAD7, KAB, and BACE-TS.

**Fig. 4:**
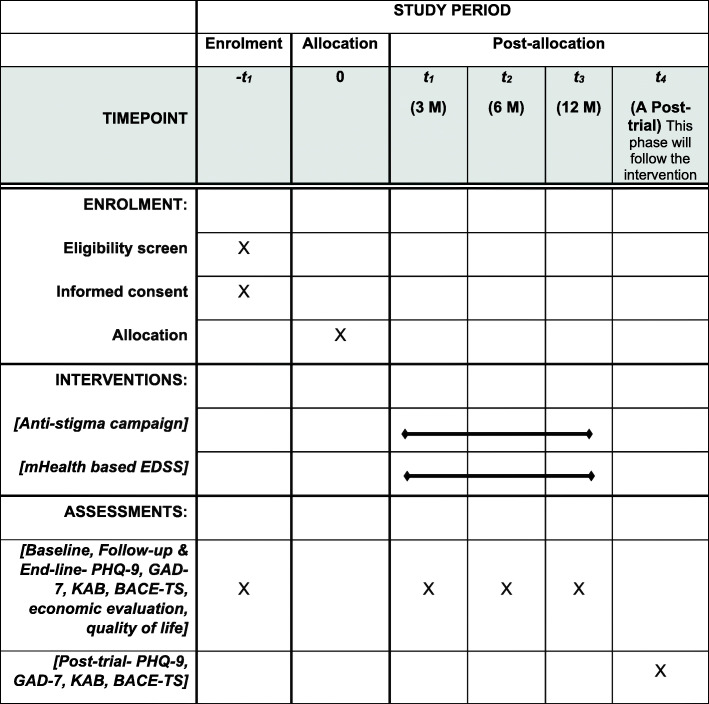
Study participant time schedule of enrolment, interventions and assessments

The SPIRIT 2013 statement, 33-item checklist and figure [36] were used to schematically represent study participants’ timeline of enrolment, eligibility screening, allocation, intervention and assessments at five timepoints (please see Fig. 4) and to guide the overall standards of a cRCT.

### Primary outcomes

There are two primary outcomes corresponding to the study hypothesis.

1. The mean difference in PHQ-9 scores at 12 months will be assessed in the high-risk cohort.
2. The difference in mean behaviour scores at 12 months using the Mental Health Knowledge, Attitude and Behaviour (KAB) scale will be analysed in the combined high-risk and non-high-risk cohort.

### Secondary outcomes

1. Remission (defined as all three of the following: PHQ-9 < 5, GAD-7 < 5 and suicide risk score < 2) in the high-risk cohort at 12 months.
2. GAD-7 scores in the high-risk cohort at 12 months
3. PHQ9 scores in the high-risk cohort at 6 months
4. GAD7 scores in the high-risk cohort at 6 months.
5. The proportion at high risk of CMDs at end of study who have visited a doctor at least once in the previous 12 months in the high-risk cohort
6. The difference in mean stigma scores at 12 months compared to baseline in the combined non-high-risk and high-risk cohort
7. The difference in mean knowledge and attitude scores and change in stigma perceptions at 12 months compared to baseline in the combined non-high-risk and high-risk cohort

### Statistical considerations

Assuming a prevalence of 4% of adults with or at high risk of CMDs, we anticipate that around 165,000 adults in total (i.e. 3750 per PHC) will need to be screened at baseline to achieve the required sample size.

In the high-risk cohort, sample sizes of 1936 in intervention and 1936 in control (total 3872), obtained by sampling 22 clusters with an average of 88 subjects each in intervention group and 22 clusters with an average of 88 subjects each in control group at the end of trial, will provide 90% power to detect a standardised mean difference of 0.4 in PHQ-9. The sample size is based on pilot study estimates [5, 37] and ICC. The sample size is based on the primary outcome. These calculations assume an intra-class correlation coefficient (ICC) of 0.15, a coefficient of variation of cluster sizes of 0.65 and a two-sided significance level of 0.05. Power Analysis and Sample Size System (PASS) software licensed by NCSS has been used for sample size estimation.

In the non-high-risk cohort, although the primary outcome will be reported for the combined high-risk and non-high-risk cohort, the study is powered on each sub-group. Sample sizes of 1936 in intervention and 1936 in control (total 3872), obtained by sampling 22 clusters with an average of 88 subjects each in intervention group and 22 clusters with an average of 88 subjects each in control group at the end of trial, will provide > 90% power to detect a standardised mean difference of 0.3 in mean behaviour scores between the intervention and control arms in each cohort. Assuming a mean behaviour score of 2 (SD 1) at baseline, and a 20% relative improvement in the control group (score of 1.6) by 12 months based on pilot and published data [37], this corresponds to a 35% improvement in the intervention group (score of 1.3) and a between-group difference of 0.3 points. This assumes a conservative ICC of 0.05 (0.01 in pilot and 0.04 in similar studies) [37], and a 2-sided significance level of 0.05.

To allow for 20% loss to follow-up during the intervention, at least 110 individuals per PHC in the high-risk cohort and at least 110 individuals per PHC in the non-high-risk cohort will be recruited.

Primary analyses will be conducted at the participant level using either random-effect models or generalised estimating equations adjusted for PHC clustering. For the primary outcome, the PHQ-9 score at 12 months, mean differences will be assessed using a linear regression including the intervention and the baseline PHQ-9 score as fixed effects and the cluster (PHC) as a random effect. If using generalised estimating equations, the effect of clustering will be accounted for using a repeated cluster effect with a compound-symmetry variance-covariance structure. The intervention effect will be estimated as the adjusted mean difference and corresponding 95% confidence interval. Subgroup analyses will be conducted according to PHC-level (size, location and health service characteristics) and patient-level characteristics (demographic factors and clinical factors, e.g. depression severity at baseline). Subgroup analyses will also be conducted to assess the difference in mean behaviour scores on KAB, at 12 months, separately for high-risk and non-high-risk cohort. Binary outcomes will be analysed similarly but using log-binomial or logistic regression in place of linear regression. A pre-specified analysis plan including sensitivity analyses, potential covariate adjustments, analyses for secondary endpoints and detailed assumptions (e.g. missing data handling) will be developed prior to unblinding and database lock.

### Economic evaluation

The economic evaluation will have a trial-based component and a modelled evaluation of long-term costs and outcomes. The economic evaluation will be conducted from both health system as well as societal perspectives. The study will collect information on health care utilisation and out-of-pocket expenditures incurred during the trial for outpatient visits, inpatient care and medications as well as lost productivity. Intervention costs will be based on salaries, training and equipment related to delivering the intervention. A trial-based incremental cost effectiveness ratio (ICER) will be determined by average differences in the number of remissions between study arms as well as average differences in utility observed between study arms using the EQ. 5D questionnaire [38]. If the intervention shows a significant difference in primary outcomes, a decision-analytic model will enable lifetime cost; long-term morbidity, quality of life and survival; and quality-adjusted life years gained to be simulated. Sensitivity analyses will be conducted to determine the robustness of the assumptions used for discount rates, intervention costs, effectiveness and other relevant model parameters used to estimate cost-utility under different scenarios. Cost-effectiveness acceptability curves will be generated to show the probability of cost-effectiveness of the intervention at a range of willingness to pay thresholds. If no significant difference is found for the primary outcomes, a cost-minimisation analysis will be performed. This comprehensive, multi-perspective economic evaluation will better inform policy makers of the resource consequences and associated benefits of implementing this programme to scale.

### Process evaluation

Normalisation process theory (NPT) [39], behaviour change theory [40] and MRC Framework for evaluating complex interventions [41] will inform the process evaluation. This will help identify local contextual factors that promote and inhibit the incorporation of a complex intervention into routine practice. This is a critical aspect of the project as it will build on the trial findings to assess scalability of the model. A case study approach will be taken, in which a purposive sample of PHC clusters will be selected to maximise variation in characteristics such as size, urban/rural, health service and baseline performance for the primary outcome. Based on prior studies, we expect around 8 PHC cases (with 24 villages) will provide enough breadth to capture a diversity of experiences with the intervention whilst allowing us to acquire a deep understanding of implementation processes within each case. A mixed methods approach will be employed to understand effective intervention strategies and significant intervention components that go into the SMART Mental Health programme. Four data sources will be used: site-specific analyses of trial data; usage analytics of the applications; quantitative satisfaction and feasibility surveys using the standardised system usability scale [42]; and in-depth, semi-structured interviews with doctors, ASHAs and community members toward the end of the study. Survey and interview instruments will be iteratively developed, guided by our overarching theories, and tested prior to implementation.

### Ethical considerations

The study is approved by the George Institute for Global Health India and All India Institute of Medical Sciences (AIIMS), New Delhi Institutional Ethics Committee (IEC). The Clinical Trial Registry of India number (CTRI/2018/08/015355) has been obtained from the National Institute of Medical Statistics, Indian Council of Medical Research (ICMR). The trial was registered on 16 August, 2018, but no patients have been recruited yet in the trial (due to the COVID crisis recruitment has been postponed to later this year) (http://ctri.nic.in/Clinicaltrials/showallp.php?mid1=23254&EncHid=&userName=CTRI/2018/08/015355). Approval of the SMART Mental Health programme from the Health Ministry’s Screening Committee (HMSC), ICMR has also been received. Letters of support from the Director General of Health Services (DGHS) of Andhra Pradesh and Haryana state governments as well as permission letters from Chief Medical Officers/Civil Surgeons at the district health department level have been granted. Preliminary discussions about the study and interventions with each village Panchayat, PHC doctors and ASHAs were conducted during the initial village mapping and household listing exercises. Informed consent will be obtained from all participants by ASHAs at the time of screening for high-risk populations and by the trained interviewers prior to administering the detailed questionnaire at baseline. Any individual identified at imminent risk of suicide at baseline will be advised to seek professional help from a doctor/psychiatrist immediately whose contact details would be provided. All data collection and reporting will be compliant with national privacy laws. Independent study monitoring will occur for a subset of participants. Data will be de-identified, stored on George Institute India servers, and held in strict compliance with Good Clinical Practice guidelines using our standard operating procedures for data security, confidentiality, backup, and audit trails. As required, all raw data and any derived datasets will be preserved for at least 10 years from study completion. Any data required to support the protocol can be supplied on request and the datasets analysed during the current study are available from the corresponding author on reasonable request. This trial does not involve collecting biological specimens for storage.

### Adverse event reporting

Serious adverse events will be captured after the start of the intervention phase. This will be defined as death due to any cause in the intervention or control arm, hospitalisation due to psychiatric disorders or a history of self-harm or attempted suicide during the intervention period. These events will be captured using a standardised case report form and reported to an independent Data Safety Monitoring Committee and Ethics Committee at interim analysis and at end of study.

### Programme milestones and trial status

Ethical approval, site approvals and modification of site materials for northern India were completed at month 12 after study commencement. Screening of the eligible adult population and baseline interviews of study participants will be completed by month 24. Randomisation of PHCs into intervention and control arm, follow-up interviews will be completed by month 36. Post-trial intervention, analysis and study close out and reporting is expected to be completed by month 48. The protocol version is 8 dated 21st April 2020. Recruitment for the north Indian site (Haryana) began from 21 September 2020 and will be complete by 20 September 2021. The recruitment for the south Indian site (Andhra Pradesh) will tentatively begin from 15th November 2020 and will complete by 14th November 2021.

## Discussion

The SMART Mental Health programme involves testing an anti-stigma campaign and an EDSS platform that allows for identification, diagnosis and treatment of CMDs. The key elements are, therefore, to increase community awareness of CMDs, and task sharing and strengthening skills of existing primary healthcare workers. It is underpinned by the World Health Organizations’ (WHO’s) Mental Health Gap Action Programme (mhGAP) [41] and builds on learnings from previous formative work [5, 23, 26, 37, 43, 44]. It includes a robust outcome evaluation design coupled with a process evaluation to understand how contextual factors drive adoption.

### Strengths and limitations

The programme strengths are that the intervention aligns with recommendations from the WHO’s Mental Health Action Plan 2013-20 [45] and India’s National Mental Health Policy to develop innovative strategies that strengthen existing systems [46]. It complements and builds on other research initiatives in LMICs including India [47, 48]. It actively explores the challenges of implementing a complex intervention in ‘real world’ settings, taking into consideration policy as well on the ground realities experienced by healthcare providers, and community members. The evidence generated has potential to inform decision-making for system planners on a scalable solution to increasing access to high-quality primary healthcare for CMDs. The main study limitation is that although it will be conducted in two different regions (South and North) of India, the outcomes may only be generalisable to similar cultural contexts and settings. The details of the study as per CONSORT checklist [49] are provided as Additional file 2.

### Significance

SMART Mental Health has potential to increase workforce capacity through supporting PHC doctors and ASHAs working at the grass roots, in the identification and management of CMDs. It also intends to have a sustainable impact on destigmatising mental disorders, increasing help seeking behaviour and treatment adherence among the community. If found to be effective and cost-effective, the strategy has potential to improve outcomes for many people who currently experience major access barriers to high-quality mental health care.

## Trial status

The protocol version is 8 dated 21 April 2020. Recruitment for the north Indian site (Haryana) began from 21 September 2020 and will be complete by 20 September 2021. The recruitment for the south Indian site (Andhra Pradesh) will tentatively begin from 15th November 2020 and will be complete by 14 November 2021.

## Supplementary Information

**Additional file 1.** SPIRIT 2013 Checklist: Recommended items to address in a clinical trial protocol and related documents.

**Additional file 2: Table 1.** CONSORT 2010 checklist of information to include when reporting a cluster randomised trial. **Table 2.** Extension of CONSORT for abstracts1^,^2 to reports of cluster randomised trials.

## Data Availability

Any data required to support the protocol can be supplied on request and the datasets analysed during the current study are available from the corresponding author on reasonable request.

## Abbreviations

CMDs: Common mental disorders
PHC: Primary health centre
ASHAs: Accredited social health activists
mHealth: Mobile health
EDSS: Electronic decision support system
SMART: Systematic Medical Appraisal Referral and Treatment
IEC: Information, education and communication
ANMs: Auxiliary nurse midwives
PHQ: Patient health questionnaire
GAD: Generalised anxiety disorders
mhGAP-IG: Mental Health Gap Action Programme-Intervention Guide
cRCT: Cluster randomised controlled trial
WHO: World Health Organization
KAB: Knowledge attitude and behaviour
BACE-TS: Barriers to Access to Care Evaluation-Treatment Stigma
NPT: Normalisation process theory
AIIMS: All India Institute of Medical Sciences
ICMR: Indian Council of Medical Research
HMSC: Health Ministry’s Screening Committee
DGHS: Director General of Health Services
LMIC: Low- and middle-income countries
