# Supplementary material for: An integrated community and primary healthcare worker intervention to reduce stigma and improve management of common mental disorders in rural India: protocol for the SMART Mental Health programme
Source: Trials. 2021 Mar 2;22:179. doi: 10.1186/s13063-021-05136-5 (PMC7923507; doi:10.1186/s13063-021-05136-5)
Supplement: Supplementary file 1 — Additional file 1. SPIRIT 2013 Checklist: Recommended items to address in a clinical trial protocol and related documents. [file 13063_2021_5136_MOESM1_ESM.doc]

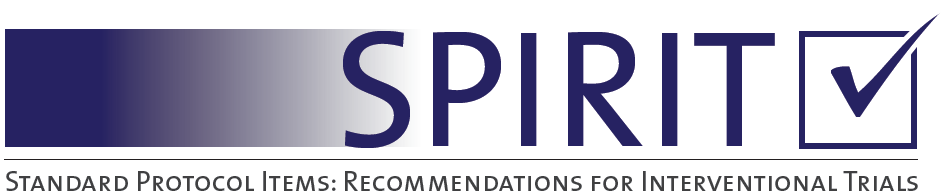


SPIRIT 2013 Checklist: Recommended items to address in a clinical trial protocol and related documents*

| Section/item | ItemNo | Description |
| --- | --- | --- |
| **Administrative information** | | |
| Title | 1 | An integrated community and primary healthcare worker intervention to reduce stigma and improve management of common mental disorders in rural India: protocol for the SMART mental health programme (Page no.1) |
| Trial registration | 2a | Clinical Trial Registry India CTRI/2018/08/015355 (Page no.4,26) |
|  | 2b | 1. Primary registry and trial identifying number: CTRI/2018/08/015355 (Page no.4,26)  2. Date of registration in primary registry: 16 August, 2018  (Page no.4,26)  3. Secondary identifying numbers: None  4. Source(s) of monetary or material support: Australian National Health and Medical Research Council (NHMRC) (Page no.30)  5. Primary sponsor: Australian National Health and Medical Research Council (NHMRC) (Page no.30)  6. Secondary Sponsor(s): None  7. Contact for public queries: Pallab Maulik, MD PhD MSc; Deputy Director and Director of Research, E-mail: [pmaulik@georgeinstitute.org.in](mailto:pmaulik@georgeinstitute.org.in); Tel: 91 40 3099 4444; The George Institute for Global Health, India; Plot No. 57, Second Floor, Corporation Bank Building, Nagarjuna Circle, Punjagutta, Hyderabad - 500 082, Telangana, India. (Page no.1)  8. Contact for Scientific Queries: PI- David Peiris, MBBS(hons) MIPH PhD FRACGP FARGP; Director, Global Primary Health Care Program; E-mail: [dpeiris@georgeinstitute.org](mailto:dpeiris@georgeinstitute.org); Tel: T +61 2 8052 4513; The George Institute for Global Health, PO Box M201 \| Missenden Rd \| NSW 2050 Australia. (Page no.3)  Pallab Maulik, MD PhD MSc; Deputy Director and Director of Research, E-mail: [pmaulik@georgeinstitute.org.in](mailto:pmaulik@georgeinstitute.org.in); Tel: 91 40 3099 4444; The George Institute for Global Health, India; Plot No. 57, Second Floor, Corporation Bank Building, Nagarjuna Circle, Punjagutta, Hyderabad - 500 082, Telangana, India. (Page no.1)  9. Public Title: Community and primary healthcare worker intervention to reduce stigma and improve management of mental disorders in rural India (SMART mental health programme) (Page no.1) |
|  |  | 10. Scientific title: An integrated community and primary healthcare worker intervention to reduce stigma and improve management of common mental disorders in rural India: protocol for the SMART mental health programme (Page no.1)  11. Countries of recruitment: India (Page no.3,12,14,15,18)  12. Health Condition(s) or Problem(s) Studied: depression, anxiety, substance use, and suicide risk (Page no.3,4,12,17)  13. Intervention(s): Anti-stigma campaign includes printed IEC materials, involving a person to talk about his/her mental illness experience, promotional, awareness and short animation videos, and staging a skit by a local theatre group. mHealth based electronic decision support system (EDSS) for primary healthcare workers based on WHO’s Mental Health Gap Action Programme- Intervention Guide (mhGAP-IG). (Page no.3,8-12)  14. Key Inclusion and Exclusion Criteria: Adults ≥18 years of age to identify high risk and non-high-risk cohort. High risk is defined as presence of at least one of the following: (1) High risk of depression based on patient health questionnaire (PHQ-9) score ≥10; (2) High risk of anxiety based on generalised anxiety disorders (GAD-7) score ≥10; and (3) Positive response (score ≥2) to the suicide risk question on the PHQ-9. Participants with either severe physical or mental ill health that would prevent regular follow-up will be excluded from the study. (Page no.14, 17)  15. Study type: Cluster randomised controlled trial (cRCT); Allocation: randomised; Intervention mode: parallel assignment; Masking: single blind (investigator and outcome assessor); Purpose: prevention and treatment. (Page no.12,18,24)  16. Date of First Enrolment: Anticipated- 1 September, 2020 (Page no.27)  17. Sample Size: Plan to enrol 7744 participants in total (Page no.22,23)  19. Recruitment Status: Pending (Page no.27)  20. Primary Outcome(s): Mean difference in patient health questionnaire (PHQ-9) scores at 12 months in the ‘high-risk’ cohort and difference in mean behaviour scores at 12 months using Mental Health Knowledge, Attitude and Behaviour (KAB) scale in the combined ‘high-risk’ and ‘non-high-risk’ cohort. (Page no.22)  21. Key Secondary Outcomes: Depression and anxiety remission rates in the ‘high-risk’ cohort at 6 and 12 months; proportion of ‘high risk’ individuals who have visited a doctor at least once in the previous 12 months; and change from baseline in mean stigma, mental health knowledge and attitude scores in the combined ‘non-high-risk’ and ‘high-risk’ cohort. (Page no.22)  22. Ethics Review: Status- approved; Original date of approval- 27 April, 2018; Date of amendment: 6 May, 2020. Name and contact details of Ethics committee(s): The George Institute Ethics Committee, The George Institute for Global Health, India, 311-312, Third Floor, Elegance Tower, Plot No. 8, Jasola District Centre  New Delhi 110025, India. (Page no.26,29)  23. Completion date: 31 May, 2022 (Page no.27) |
|  |  | 24. Summary Results: As the recruitment of study participants and the intervention is pending, there are no results to share.  24. IPD sharing statement:  Plan to share IPD: YES  The data will be free for sharing once the primary outcomes are in public domain. This will be done on specific request to the PIs and will need to adhere to the institutional guidelines on data sharing which follow ICMJE guidelines. (Page no.26,27) |
| Protocol version | 3 | Issue Date: 21 April, 2020  Protocol Amendment Number: 08  Author(s): MD, PKM, SK, AK, SD, AB, LB, DPr, BE, SC, AP, DP  (Page no.27) |
| Funding | 4 | Australian National Health and Medical Research Council (NHMRC)  (Page no.30) |
| Roles and responsibilities | 5a | DP, PKM and AP conceptualised the study. PKM leads the implementation of the trial in India along with MD, SD, SKl, AK and AM. LB and AB contributed to the statistical aspects of the study. BE offered analytical expertise on the design of the economic evaluation. Each author has critically reviewed, commented and approved the final study protocol. (Page no.31) |
|  | 5b | Trial Sponsor: Australian National Health and Medical Research Council (NHMRC); Sponsor’s Reference: APP1143911; Contact name: Pru Glasson; Address: 16 Marcus Clarke St,  Canberra ACT 2601; Telephone: +61 7 30001234; Email: [nhmrc@nhmrc.gov.au](mailto:nhmrc@nhmrc.gov.au) (Page no.31) |
|  | 5c | This funding source had no role in the design of this study and will not have any role during its execution, analyses, interpretation of the data, or decision to submit results. (Page no.31) |
|  | 5d | 2.4 Composition, roles, and responsibilities of groups overseeing the study  Coordinating Centre: Conduct of the study, organisation of steering committee meetings, budget and manpower administration, contractual issues with individual sites.  Study coordinating committee: Study planning, organisation of steering committee meetings, responsible for trial master data, Assistance with independent ethics committee applications, data collection and verification, randomisation.  Steering Committee: Agreement of final protocol, reviewing progress of study and if necessary recommend protocol modifications.  Data Safety Monitoring Committee: Review safety data on a periodic basis, recommend the steering committee to either stop or amend project implementation based on safety findings.  Data Management Team: data collection and verification, randomisation, data analyses.  Statistical Team: analyses of outcome data  (Page no.27,31) |
| Introduction |  |  |
| Background and rationale | 6a | Around 1 in 7 people in India are impacted by mental illness. The treatment gap for people with mental disorders is as high as 75-95%. Health care systems, especially in rural regions in India face substantial challenges to address these gaps in care, and innovative strategies are needed. (Page no.3,5) |
|  | 6b | The control arm will consist of both high-risk and non high-risk individuals as the intervention arm but will only receive enhanced usual care. (Page no.14) |
| Objectives | 7 | Address mental healthcare access barriers and lead to significant improvements in community behaviours toward mental disorders; and improvements in the proportion of adults at high risk of mental disorders achieving remission for depression, anxiety and suicide risk. (Page no.12) |
| Trial design | 8 | Cluster randomised controlled trial (cRCT); Allocation: stratified by remission rate and population size and then central 1:1 randomisation; Intervention mode: parallel assignment; Masking: single blind (investigator and outcome assessor); (Page no.12, 13) |
| Methods: Participants, interventions, and outcomes | | |
| Study setting | 9 | 44 Primary Health Centres and 133 villages in West Godavari district of Andhra Pradesh, and Faridabad and Palwal districts of Haryana in India. (Page no.15,16) |
| Eligibility criteria | 10 | Adults ≥18 years of age to identify high risk and non-high-risk cohort. High risk is defined as presence of at least one of the following: (1) High risk of depression based on patient health questionnaire (PHQ-9) score ≥10; (2) High risk of anxiety based on generalised anxiety disorders (GAD-7) score ≥10; and (3) Positive response (score ≥2) to the suicide risk question on the PHQ-9. Participants with either severe physical or mental ill health that would prevent regular follow-up will be excluded from the study. (Page no.14, 17) |
| Interventions | 11a | Anti-stigma campaign includes printed IEC materials, involving a person to talk about his/her mental illness experience, promotional, awareness and short animation videos, and staging a skit by a local theatre group. mHealth based electronic decision support system (EDSS) for primary healthcare workers based on WHO’s Mental Health Gap Action Programme- Intervention Guide (mhGAP-IG). The two core intervention components will work simultaneously with the anti-stigma campaign having an intensive three months phase at the beginning. (Page no.3,8-12) |
|  | 11b | The Data safety Monitoring Committee will guide this, and trial will be stopped only when an increase in the proportion of participants with a suicide risk score >2 at 3 and 6 months in the intervention arm of 3 standard deviations or more (Page no.27) |
|  | 11c | The electronic decision support system used by primary health workers will allow them to track individual care and ensure better treatment adherence. An algorithm-based interactive voice response system that will send out pre–recorded messages to diagnosed patients to continue care as advised by primary healthcare workers, and similar motivational messages will be sent to primary healthcare workers to screen and follow up patients as per guidelines. (Page no.11,12) |
|  | 11d | There are no restrictions imposed in relation to any relevant concomitant care and interventions that are permitted or prohibited during the trial. (Page no.19,20) |
| Outcomes | 12 | Primary outcomes: (1) The mean difference in patient health questionnaire (PHQ-9) scores at 12 months in the ‘high-risk’ cohort, (2) The difference in mean behaviour scores at 12 months using the Mental Health Knowledge, Attitude and Behaviour (KAB) scale in the combined ‘high-risk’ and ‘non-high-risk’ cohort.  Secondary outcomes: (1) Remission (defined as all three of the following: patient health questionnaire (PHQ-9) <5, generalised anxiety disorders (GAD-7) <5 and suicide risk score <2) in the ‘high-risk’ cohort at 12 months; (2) GAD-7 scores in the ‘high risk’ cohort at 12 months; (3) PHQ9 scores in the ‘high-risk’ cohort at 6 months; (4) GAD7 scores in the ‘high-risk’ cohort at 6 months; (5) The proportion at high risk of CMDs at end of study who have visited a doctor at least once in the previous 12 months in the ‘high risk’ cohort; (6) The difference in mean stigma scores at 12 months compared to baseline in the combined ‘non-high-risk’ and ‘high-risk’ cohort; (7) The difference in mean knowledge and attitude scores and change in stigma perceptions at 12 months compared to baseline in the combined ‘non-high-risk’ and ‘high-risk’ cohort. (Page no.22) |
| Participant timeline | 13 | Data collection will take place on five occasions: prior to randomisation (Time 0); during intervention (3, 6, 12 months); and at the end of the post-trial phase. patient health questionnaire (PHQ-9), generalised anxiety disorders (GAD-7), Knowledge, Attitude, and Behaviour (KAB) and Barriers to Access to Care Evaluation- Treatment Stigma (BACE-TS) will be administered at these time points. The SPIRIT figure is included and referenced in the main body of the text of the manuscript. (Page no.20,21) |
| Sample size | 14 | In the high-risk cohort, sample sizes of 1936 in intervention and 1936 in control (total 3872), obtained by sampling 22 clusters with an average of 88 subjects each in intervention group and 22 clusters with an average of 88 subjects each in control group at the end of trial, will provide 90% power to detect a standardised mean difference of 0.4 in PHQ-9. These calculations assume an intra-class correlation coefficient (ICC) of 0.15, a coefficient of variation of cluster sizes of 0.65 and a two-sided significance level of 0.05.  In the non-high-risk cohort, although the primary outcome will be reported for the combined high and non-high-risk cohort the study is powered on each sub-group. Sample sizes of 1936 in intervention and 1936 in control (total 3872), obtained by sampling 22 clusters with an average of 88 subjects each in intervention group and 22 clusters with an average of 88 subjects each in control group at the end of trial, will provide >90% power to detect a standardised mean difference of 0.3 in mean behaviour scores between the intervention and control arms in each cohort. Assuming a mean behaviour score of 2 (SD 1) at baseline, and a 20% relative improvement in the control group (score of 1.6) by 12 months based on pilot and published data [36], this corresponds to a 35% improvement in the intervention group (score of 1.3) and a between-group difference of 0.3 points. This assumes a conservative ICC of 0.05 (0.01 in pilot and 0.04 in similar studies), and a 2-sided significance level of 0.05. (Page no.22,23) |
| Recruitment | 15 | Appropriate site selection, census of population prior to the study, engaging with communities, detailed participant information sheet, diligent follow up by field investigators (Page no.14,21) |
| **Methods: Assignment of interventions (for controlled trials)** | | |
| Allocation: |  |  |
| Sequence generation | 16a | Allocation of clusters to intervention or control arm will use a computer-generated 1:1 central allocation sequence. Random allocation will be performed using SAS PROC plan or another relevant procedure. (Page no.18) |
| Allocation concealment mechanism | 16b | To maintain blinding, the unblinded statistician will generate the actual randomisation list and share with field staff. The blinded statistician on the study will work with dummy treatment arms until end of study. The treatment related columns will not be shared with blinded statistician/ statistical team until the time of planned study unblinding. (Page no.18,24) |
| Implementation | 16c | The allocation of primary health centres to intervention or control will be done by web-based allocation; the implementation team will implement that decision on the ground. (Page no.18-20) |
| Blinding (masking) | 17a | Independent field investigators, blinded to intervention allocation, will be involved in data collection at each phase of the study. The treatment related data will not be shared with blinded statistician/ statistical team until the time of planned study unblinding. (Page no.18,24) |
|  | 17b | Only if the study is stopped on the recommendation of the DSMC, unblinding is permissible and revealing a participant’s allocated intervention during trial. (As per DSMC Terms of Reference) |
| **Methods: Data collection, management, and analysis** | | |
| Data collection methods | 18a | PHQ-9 and GAD7 are the main screening tools that will be utilised. Both these scales have been validated in India. Barriers to Access to Care Evaluation-Treatment Stigma Subscale (BACE-TS version 3) has been found to have good reliability and construct validity. Mental Health Knowledge, Attitude and Behaviour test-retest reliability and internal consistency is high. (Page no.3,10,20,21) |
|  | 18b | A minimum of three follow-up visits will be made by primary healthcare workers by visiting participants’ residence. An interactive voice response system will send out pre–recorded messages to participants to continue care. (Page no.11,12) |
| Data management | 19 | All data collection and reporting will be compliant with national privacy laws. Independent study monitoring will occur for a subset of participants. Data will be de-identified, stored on George Institute India servers, and held in strict compliance with Good Clinical Practice guidelines using our standard operating procedures for data security, confidentiality, backup, and audit trails. As required, all raw data and any derived datasets will be preserved for at least 10 years from study completion. (Page no.26,27) |
| Statistical methods | 20a | Primary analyses will be conducted at the participant level using either random-effect models or generalised estimating equations adjusted for PHC clustering. For the primary outcome, the PHQ-9 score at 12 months, mean differences will be assessed using a linear regression including the intervention and the baseline PHQ-9 score as fixed effects and the cluster (PHC) as a random effect. If using generalised estimating equations, the effect of clustering will be accounted for using a repeated cluster effect with a compound-symmetry variance-covariance structure. The intervention effect will be estimated as the adjusted mean difference and corresponding 95% confidence interval. (Page no.24) |
|  | 20b | Subgroup analyses will be conducted according to PHC-level (size, location and health service characteristics) and patient-level characteristics (demographic factors and clinical factors e.g. depression severity at baseline). Subgroup analyses will also be conducted to assess the difference in mean behaviour scores on KAB, at 12 months, separately for ‘high-risk’ and ‘non-high-risk’ cohort. Binary outcomes will be analysed similarly but using log-binomial or logistic regression in place of linear regression. (Page no.24) |
|  | 20c | A pre-specified analysis plan including sensitivity analyses, potential covariate adjustments, analyses for secondary endpoints and detailed assumptions (e.g. missing data handling) will be developed prior to unblinding and database lock. (Page no.24) |
| **Methods: Monitoring** | | |
| Data monitoring | 21a | A Data Safety and Monitoring Committee (DSMC) has been established. The DSMC is an independent committee that makes recommendations to the study and Steering Committee, and will receive technical support from the project statistical and data management teams. The DSMC members are responsible for the following:  • Safeguarding the interests of the project participants  • Assessing the efficacy and safety of the intervention throughout the project  • Monitoring the overall conduct of the project  • Providing recommendations (regarding stopping or continuing the project) to the steering committee  • Contributing to enhancing the integrity of the project  Interim analyses will be supplied, in strict confidence, to the DSMC, together with any other analyses that the committee may request. The DSMC is an advisor to the steering committee and will be responsible for promptly reviewing the project data and providing recommendations to continue, terminate or amendments to project conduct is required.  Names of chair, members and terms of reference for the DSMC are available on request from the corresponding author. (Page no.27) |
|  | 21b | In order to address safety concerns, at least one interim analysis will be conducted with results reviewed by the DSMC. The standard analysis and reporting format of the Data Monitoring Reports will be developed in agreement with the independent Statistics Group, with the support of the project statistician at The George Institute and under the direction of the DSMC. Although no formal/ binding stopping rules are suggested, an increase in the proportion of participants with a suicide risk score >2 at 3 and 6 months in the intervention arm of 3 standard deviations or more would be regarded as strong evidence of early harm. The DSMC will reveal the unblinded results to the Steering Committee if, considering both statistical and clinical issues and exercising their best clinical and statistical judgement, the unblinded results provide enough evidence that the trial treatment is on balance harmful. (Page no.27) |
| Harms | 22 | Serious adverse events will be captured after the start of the intervention phase. This will be defined as death due to any cause in the intervention or control arm, hospitalisation due to psychiatric disorders, or a history of self-harm or attempted suicide during the intervention period. These events will be captured using a standardised case report form and reported to an independent Data Safety Monitoring Committee and Ethics Committee at interim analysis and at end of study. (Page no.27) |
| Auditing | 23 | Research Management and Operations Management teams at the institute who are independent of the investigators run monthly checks on each project and if any issues are flagged more detailed discussions are held. (Internal institutional document) |
| Ethics and dissemination | | |
| Research ethics approval | 24 | This protocol and the template informed consent forms contained in Appendix II will be reviewed and approved by the George Institute for Global Health India and All India Institute of Medical Sciences (AIIMS), New Delhi Institutional Ethics Committee (IEC). The protocol, site-specific participant information sheet and informed consent forms (local language and English versions), study tools and other requested documents — and any subsequent modifications — also will be reviewed and approved by the Institutional Ethics Committee (IEC). (Page no.26) |
| Protocol amendments | 25 | Any modifications to the protocol which may impact on the conduct of the study, potential benefit of the patient or may affect patient safety, including changes of study objectives, study design, patient population, sample sizes, study procedures, or significant administrative aspects will require a formal amendment to the protocol. Such amendment will be agreed upon by study coordinating committee and approved by the steering committee and approved by the Institutional Ethics Committee (IEC). This will be further amended in the national Clinical Trial Registry of India number, National Institute of Medical Statistics, Indian Council of Medical Research (ICMR). (Page no.26,27) |
| Consent or assent | 26a | Informed consent will be obtained from all participants by primary healthcare workers at the time of screening for high-risk populations and by the trained interviewers prior to administering the detailed questionnaire at baseline and follow-up assessments. Primary healthcare workers and interviewers will discuss the study and intervention with participants in light of the information provided in the participant information sheet. Participants will then be able to have an informed discussion with them and written consent from participants will be obtained from participants willing to participate in the study. All participant information sheet and consent forms have been translated into Hindi and Telugu. (Page no.26) |
|  | 26b | This trial does not involve collecting biological specimens for storage (Page no. 27) |
| Confidentiality | 27 | All data collection and reporting will be compliant with national privacy laws. Data will be de-identified, stored on George Institute India servers, and held in strict compliance with Good Clinical Practice guidelines using our standard operating procedures for data security, confidentiality, backup, and audit trails. (Page no.26.27) |
| Declaration of interests | 28 | There are no financial and other competing interests for principal investigators for the overall trial and each study site. |
| Access to data | 29 | The Data Management team will oversee the intra-study data sharing processes, with input from the Statistical team. All investigators (both in Australia and host country India) will be given access to the cleaned data sets. Project data sets will be stored on George Institute India servers. To ensure confidentiality, data shared with project team members will be blinded of any identifying participant information. Any data required to support the protocol can be supplied on request (Page no.14,20,26,27.) |
| Ancillary and post-trial care | 30 | All studies have insurance coverage and if anyone claims any compensation, then it needs to be first approved by the ethics committee as per national regulations, and then the insurance company, the research institute and the claimant will discuss it further. |
| Dissemination policy | 31a | Most peer-reviewed publications will be done using open access publications. Lay documents for public consumption will be developed and distributed. Following completion of the study, results will be shared with all key stakeholders through face-to-face meetings, brochures, policy briefs, presentations and publications. Policy symposia will be held to disseminate study findings and to engage government, non-government and industry stakeholders to discuss transitioning the intervention to a non-research environment. Although the shape of these discussions will be influenced by the evaluation findings, the goal will be to identify the business models for implementing the intervention into routine service delivery. (As per protocol document) |
|  | 31b | A separate committee will be responsible for ensuring standard practices for dissemination of the results either via peer reviewed publications, policy documents, or media releases. Wherever necessary, expert opinion would be sought to guide that group on appropriateness of the content. Professional writers will not be involved. (As per protocol document) |
|  | 31c | The protocol paper is being published and will be the publicly accessible document. It captures all details of the protocol. The data will be free for sharing once the primary outcomes are in public domain. This will be done on specific request to the PIs and will need to adhere to the institutional guidelines on data sharing which follow ICMJE guidelines. The datasets analysed during the current study are available from the corresponding author on reasonable request. (Page no.26.27) |
| Appendices |  |  |
| Informed consent materials | 32 | Sample participant information sheet and consent form. The full forms are not shared here but can be obtained from the corresponding author on request.  **Study Title: The Systematic Medical Appraisal, Referral and Treatment (SMART) Mental Health Programme**  **Participant Information Sheet for Community Participant (Interviewer)**  1.Introduction  You are invited to take part in the study entitled The Systematic Medical Appraisal, Referral and Treatment (SMART) Mental Health Programme.”  This Participant Information Statement contains detailed information about the study. Its purpose is to explain to you as openly and clearly as possible all the procedures involved in this study.  2. Purpose and Background  The George Institute for Global Health aims to develop and evaluate a system to identify individuals with common mental disorders (CMD) – stress, depression, suicide risk, and effectively manage them in their local settings. |
|  |  | 3. Procedures  The following are involved:  1) A screening for CMDs among adults was done by the ASHAs.  4. Risks or discomforts on participation  There is minimal risk to participants during participation in this study.  5. Possible benefits on participation  The study would provide you with an understanding of whether you suffer from psychological stress or a CMD.  6. Privacy, Confidentiality and Disclosure of Information  All the information collected from you for the study will be kept strictly confidential.  7. Participation is Voluntary  Participation in this research study is voluntary.  8. What if something goes wrong?  The George Institute for Global Health holds insurance policies which apply to this study.  9. Contact details  When you have read this information, the interviewers will discuss it with you further and will answer any questions you may have. |
|  |  |  |
|  |  | Contact person:  Details provided in the actual form (Not included in SPIRIT template)    Principal Investigator:  Details provided in the actual form (Not included in SPIRIT template)  Statement of approval:  This study has received ethical approval from The George Institute Ethics Committee (TGIEC), New Delhi.  Any person with concerns or complaints about the conduct of the research study can contact  TGIEC Ethics Committee:  Member Secretary Institutional Ethics Committee,  The George Institute Ethics Committee  311-312, Third Floor, Elegance Tower,  Plot No 8, Jasola District Centre,  New Delhi, 110025  Telephone: 011- 41588091  Email: membersecretary@georgeinstitute.org.in |
|  |  | **Participant ID:**  **The Systematic Medical Appraisal, Referral and Treatment (SMART) Mental Health Programme**  **Participant Consent Form for Community Participant Study Site**  Please initial the box if you agree with the following:   I have read the Participant Information Sheet.  **Participant**  Signature/  Thumb Impression:  Date: ..........................  Name: .........................  **Witness***  Signature: …………  Date: ………………  Name: ………………  *Caretaker/ Well-wisher  **Person taking the Consent**  Signature: …………  Date: ………………  Name: ………………  Signature of Principal Investigator:  Details provided in the actual form (Not included in SPIRIT template) |
| Biological specimens | 33 | This does not apply here as the study is a community based non-clinical trial and will not collect any biological specimen data. |

*It is strongly recommended that this checklist be read in conjunction with the SPIRIT 2013 Explanation & Elaboration for important clarification on the items. Amendments to the protocol should be tracked and dated. The SPIRIT checklist is copyrighted by the SPIRIT Group under the Creative Commons “[Attribution-NonCommercial-NoDerivs 3.0 Unported](http://www.creativecommons.org/licenses/by-nc-nd/3.0/)” license.
